# A quantitative study of neurochemically defined populations of inhibitory interneurons in the superficial dorsal horn of the mouse spinal cord

**DOI:** 10.1016/j.neuroscience.2017.08.044

**Published:** 2017-11-05

**Authors:** Kieran A. Boyle, Maria Gutierrez-Mecinas, Erika Polgár, Nicole Mooney, Emily O'Connor, Takahiro Furuta, Masahiko Watanabe, Andrew J. Todd

**Affiliations:** a Institute of Neuroscience and Psychology, College of Medical, Veterinary and Life Sciences, University of Glasgow, Glasgow G12 8QQ, UK; b Department of Morphological Brain Science, Graduate School of Medicine, Kyoto University, Kyoto 606-8501, Japan; c Department of Anatomy, Hokkaido University School of Medicine, Sapporo 060-8638, Japan

**Keywords:** AAV, adeno-associated virus, DAPI, 4′,6-diamidino-2-phenylindole, eGFP, enhanced green fluorescent protein, nNOS, neuronal nitric oxide synthase, NPY, neuropeptide Y, PKCγ, protein kinase Cγ, PPD, preprodynorphin, tdTom, tdTomato, TSA, tyramide signal amplification, neuropeptide Y, neuronal nitric oxide synthase, dynorphin, galanin, inhibitory interneuron

## Abstract

Around a quarter of neurons in laminae I–II of the dorsal horn are inhibitory interneurons. These play an important role in modulating somatosensory information, including that perceived as pain or itch. Previous studies in rat identified four largely non-overlapping neurochemical populations among these cells, defined by expression of galanin, neuropeptide Y (NPY), neuronal nitric oxide synthase (nNOS) or parvalbumin. The galanin cells were subsequently shown to coexpress dynorphin. Several recent studies have used genetically modified mice to investigate the function of different interneuron populations, and it is therefore important to determine whether the same pattern applies in mouse, and to estimate the relative sizes of these populations. We show that the neurochemical organization of inhibitory interneurons in mouse superficial dorsal horn is similar to that in the rat, although a larger proportion of these neurons (33%) express NPY. Between them, these four populations account for ∼75% of inhibitory cells in laminae I–II. Since ∼25% of inhibitory interneurons in this region belong to a novel calretinin-expressing type, our results suggest that virtually all inhibitory interneurons in superficial dorsal horn can be assigned to one of these five neurochemical populations. Although our main focus was inhibitory neurons, we also identified a population of excitatory dynorphin-expressing cells in laminae I–II that are largely restricted to the medial part of the mid-lumbar dorsal horn, corresponding to glabrous skin territory. These findings are important for interpretation of studies using molecular-genetic techniques to manipulate the functions of interneuron populations to investigate their roles in somatosensory processing.

## Highlights

• Neurochemistry of lamina I–II inhibitory neurons in mouse is similar to that in rat.
• Five neurochemical classes account for all lamina I–II inhibitory neurons in mouse.
• Excitatory dynorphin cells are largely restricted to glabrous skin territory.

## Introduction

The spinal dorsal horn receives sensory input from a wide variety of primary afferents, including nociceptors, pruritoceptors, thermoreceptors and low-threshold mechanoreceptors, and these terminate in a highly organized pattern within specific laminae (Todd, 2010, Todd, 2017, Abraira and Ginty, 2013, Braz et al., 2014). The incoming sensory information is processed through complex synaptic circuits before being transmitted via projection neurons to the brain (for conscious perception), as well as to neurons involved in spinal reflex pathways. The main components involved in these modulatory circuits are local interneurons, which are extremely numerous, and are thought to constitute around 99% of the neurons within the dorsal horn (Abraira and Ginty, 2013). Dorsal horn interneurons can be divided into two broad functional classes: inhibitory neurons, which use GABA and/or glycine as their principal fast transmitter, and excitatory (glutamatergic) neurons (Todd et al., 2003, Yasaka et al., 2010, Zeilhofer et al., 2012). Quantitative studies in the mouse have shown that the inhibitory interneurons account for around one quarter of the neurons in lamina I–II and ∼40% of those in lamina III. These cells are known to have an important role in suppressing pain and itch, and loss of this function is thought to contribute to pathological pain states (Yaksh, 1989, Coull et al., 2003, Sandkuhler, 2009, Kardon et al., 2014, Foster et al., 2015, Petitjean et al., 2015).

The inhibitory interneurons can be divided into specific classes, based on the expression of certain neuropeptides and proteins (Todd, 2010, Todd, 2017, Braz et al., 2014), and there is increasing evidence that these neurochemical classes correspond to functional populations. For example, they differ in laminar location, which is likely to reflect specific patterns of primary afferent input to each class, and in their responses to noxious stimuli (Polgar et al., 2013b). In 2011, we identified four largely non-overlapping populations among the inhibitory interneurons in laminae I–III of the rat spinal cord, defined by expression of galanin, neuropeptide Y (NPY), neuronal nitric oxide synthase (nNOS) and parvalbumin (Tiong et al., 2011). We subsequently reported that most of the galanin-containing cells also express the opioid peptide dynorphin, although dynorphin was also found in some excitatory interneurons (Sardella et al., 2011a). Between them, these four populations (galanin/dynorphin, NPY, nNOS and parvalbumin) were thought to account for just over half of the inhibitory interneurons in laminae I–II (Todd, 2017).

Since then, several studies have made use of these neurochemical features to manipulate the activity of neuronal populations in genetically modified mouse lines and test the effects on pain and itch behavior (Duan et al., 2014, Bourane et al., 2015, Foster et al., 2015, Petitjean et al., 2015). These studies have provided important insights into the functions of inhibitory interneuron populations. However, interpretation of findings from studies of this type is complicated, firstly because proteins of interest may be transiently expressed by certain neurons in the dorsal horn (Duan et al., 2014, Bourane et al., 2015, Gutierrez-Mecinas et al., 2017), meaning that behavioral changes cannot necessarily be attributed to the intended neuronal populations. In addition, single neurochemical/genetic markers are often expressed by more than one functional population. For example, nNOS, parvalbumin and dynorphin are also found in significant numbers of excitatory interneurons. Finally, although similar neurochemical populations to those that we defined in the rat can be identified in the mouse dorsal horn, less is known about the sizes of these populations, or the extent to which they overlap. Indeed, there appear to be some species differences, because unlike the situation in the rat, nNOS and galanin show significant overlap in the mouse (Iwagaki et al., 2013, Kardon et al., 2014).

The main aim of this study was to define and quantify neurochemical populations among the inhibitory interneurons in the mouse superficial dorsal horn, and to determine what proportion of the inhibitory neurons they account for. This information will be of importance for interpreting studies in which specific interneuron populations are targeted in genetically modified mice.

## Experimental procedures

### Animals

All experiments were approved by the Ethical Review Process Applications Panel of the University of Glasgow, and were performed in accordance with the European Community directive 86/609/EC and the UK Animals (Scientific Procedures) Act 1986.

Nine adult C57Bl/6 mice of either sex (20–28 g) were deeply anesthetized with pentobarbitone (30 mg i.p.) and perfused through the left cardiac ventricle with fixative consisting of 4% freshly depolymerized formaldehyde in phosphate buffer. Lumbar spinal cord segments were removed and stored at 4 °C for 2 h in the same fixative. Tissue from these mice was processed for immunocytochemistry (see below) to reveal interneurons belonging to different neurochemical populations.

As an additional way of identifying neurons that express dynorphin, we also analyzed tissue from mice in which Cre recombinase had been knocked into the prodynorphin gene (Pdyn^Cre^) (Krashes et al., 2014). Pdyn^Cre^ mice were crossed with the Ai9 reporter line (Jackson Laboratory; Stock number 007909), in which Cre-mediated excision of a STOP cassette drives expression of the red fluorescent protein tdTomato (tdTom). The resulting mice (Pdyn^Cre^;Ai9) should have tdTom in all neurons that have expressed dynorphin at any stage during development. Four male Pdyn^Cre^;Ai9 mice (20–25 g) were anesthetized and perfused with fixative, and spinal cord tissue was removed and processed as described above.

To distinguish neurons that continue to express dynorphin past the early postnatal period, we performed intraspinal injections of adeno-associated (AAV) virus (serotype 1) carrying a conditional (Cre-dependent) enhanced green fluorescent protein (eGFP) expression cassette (AAV.flex.eGFP; Penn Vector Core, Philadelphia, PA USA) (Gutierrez-Mecinas et al., 2017). The virus encodes the inverted sequence for eGFP between pairs of heterotypic LoxP sites with antiparallel orientation (Atasoy et al., 2008). The rationale for this approach is that in infected dorsal horn neurons that express Cre at the time of injection, there will be permanent reversal of the coding sequence, resulting in expression of eGFP. Three Pdyn^Cre^;Ai9 mice (either sex, 15–17 g, aged P30–44) were anesthetized with isoflurane and received two injections of AAV.flex.eGFP (each 1.7 × 10^9^ GC in 300 nl) into the dorsal horn on the right side, as described previously (Gutierrez-Mecinas et al., 2017). The injections were made on either side of the T13 vertebra, at ∼300 μm from the midline and at a depth of 300 μm below the pial surface. The wound was closed and the animals allowed to recover with appropriate post-operative analgesia. After a survival period of 13 or 14 days, they were re-anesthetized and fixed by perfusion, and tissue was processed as described above.

### General features of immunostaining and confocal microscopy

Lumbar spinal cord segments were cut into 60-μm-thick transverse sections with a vibrating blade microtome. These were immersed for 30 min in 50% ethanol to enhance antibody penetration and reacted for multiple-labeling immunofluorescence staining as described previously (Gutierrez-Mecinas et al., 2014, Gutierrez-Mecinas et al., 2016, Cameron et al., 2015, Ganley et al., 2015). Details of the antibodies used in this study, including the sources and concentrations, are provided in Table 1. Unless otherwise stated, the sections were incubated for 3 days at 4 °C in primary antibodies diluted in PBS that contained 0.3 M NaCl, 0.3% Triton X-100 and 5% normal donkey serum, and then overnight in species-specific secondary antibodies (Jackson Immunoresearch, West Grove, PA, USA) that were raised in donkey and conjugated to Alexa 488, Alexa 647, Rhodamine Red, Pacific Blue or biotin. All secondary antibodies were diluted 1:500 (in the same diluent), apart from those conjugated to Rhodamine Red and Pacific Blue, which were diluted 1:100 and 1:200, respectively. Biotinylated secondary antibodies were detected either with Pacific Blue conjugated to avidin (1:1000; Life Technologies, Paisley, UK) or with a tyramide signal amplification (TSA) method (TSA kits tetramethylrhodamine NEL702001 or Cy5 NEL705A001, PerkinElmer Life Sciences, Boston, MA, USA). The TSA reaction was used to detect antibody against preprodynorphin (PPD), as this method can be used to reveal the cell bodies of dorsal horn neurons that express dynorphin (Sardella et al., 2011a, Kardon et al., 2014). Sections were mounted in anti-fade medium and stored at −20 °C.

**Table 1:** Antibodies used in this study

Antibody	Species	Catalog no	Dilution	Source
NPY	Rabbit	T-4070	1:1000	Peninsula
Galanin	Rabbit	T-4334	1:1000	Peninsula
PPD	Guinea pig		1:5000*	T Furuta
PPD	Rabbit		1:10,000*	T Furuta
nNOS	Goat		1:1000	M Watanabe
Parvalbumin	Guinea pig		1:2500	M Watanabe
sst_2A_	Guinea pig	SS870	1:2000	Gramsch Laboratories
Pax2	Rabbit	716000	1:1000	Life Technologies
NeuN	Mouse	MAB377	1:500	Merck
PKCγ	Rabbit	sc211	1:1000	Santa Cruz Biotechnology

* For tyramide signal amplification.

Immunostained sections were scanned with a Zeiss LSM710 confocal microscope equipped with Argon multi-line, 405-nm diode, 561-nm solid state and 633-nm HeNe lasers. Confocal image stacks, generally consisting of at least 30 optical sections (with a z-separation of 1 μm), were obtained from the entire mediolateral extent of the dorsal horn through a 40× oil-immersion lens (numerical aperture 1.3) with the confocal aperture set to 1 Airy unit or less. The resulting z-stacks were analyzed with Neurolucida for Confocal software (MBF Bioscience, Williston, VT, USA). Most of the analysis was carried out on the superficial dorsal horn (laminae I and II). The border between laminae II and III was identified either based on the relatively low packing density of neurons in the inner half of lamina II (Rexed, 1952, Molander et al., 1984, Todd et al., 1998) or by staining for protein kinase Cγ (PKCγ), which labels a dense plexus of dendrites that extends ventrally as far as the lamina II/III border (Hughes et al., 2003). In some cases, lamina III was included in the analysis and its ventral border was identified by the reduction in neuronal packing density seen in lamina IV. Quantitative analyses were generally performed using a modification (Polgár et al., 2004) of the disector method (Sterio, 1984) on sections that had been immunostained for NeuN, and in some cases counterstained with the nuclear stain 4′,6-diamidino-2-phenylindole (DAPI). The reference and look-up sections were set between 10 and 20 μm apart, and all neurons for which the bottom surface of the soma (or nucleus, in sections stained with DAPI) lay between these optical sections were included in the analysis. In cases where we estimated the density of cells per unit length of spinal cord, a correction factor was applied to compensate for tissue shrinkage in the z-axis (Polgár et al., 2004, Polgar et al., 2013a). This factor was the measured z-depth of the section as seen on the confocal microscope, divided by the original section thickness (60-μm).

### NPY-expressing interneurons

Sections from the L4 segment of four mice were reacted with antibodies against NPY, PPD, sst_2A_ and NeuN. These were used to estimate the proportion of neurons in laminae I–III that are NPY-immunoreactive, to determine the extent of co-localization between NPY and dynorphin, and to test whether cells that contained both neuropeptides expressed sst_2A_. Three or four sections from each mouse were scanned and analyzed with the disector method. The NeuN channel was initially used to mark the location of neurons in the disector sample that were located in laminae I–II and in lamina III. The other channels were then viewed and the expression of PPD, NPY and/or sst_2A_ in each selected neuron was recorded.

It has been reported that all NPY-immunoreactive neurons in laminae I–III of the rat dorsal horn are also GABA-immunoreactive, and are therefore inhibitory interneurons (Rowan et al., 1993). To test whether this is also the case in the mouse we immunostained sections from the L4 segments of three mice to reveal NPY and the transcription factor Pax2. Pax2 is restricted to inhibitory interneurons, and has been shown to be expressed by all of the inhibitory cells in laminae I–III (Kardon et al., 2014, Foster et al., 2015, Larsson, 2017). Because both antibodies are raised in the rabbit, we used a sequential staining method. Sections were initially incubated in the Pax2 and NeuN antibodies, which were detected with secondary antibodies conjugated to Alexa 488 and Alexa 647, respectively. They were then reacted with rabbit anti-NPY which was revealed with Rhodamine Red. Finally, they were counterstained with DAPI. Although the Pax2^+^ nuclei were labeled with both Alexa 488 and Rhodamine Red, this staining was restricted to the cell nucleus, and was clearly distinct from cytoplasmic staining for NPY (which was labeled only with Rhodamine Red). Two sections from each mouse were scanned and examined with the Neurolucida for Confocal. We searched for neurons in laminae I–III that showed NPY immunoreactivity in their perikaryal cytoplasm and looked for the presence of Pax2 staining in the nucleus.

We also examined sections that had been reacted with antibodies against NPY, nNOS and parvalbumin, in order to assess any overlap between these three neurochemical markers. Two sections from each of three mice were scanned and analyzed. We selected parvalbumin-positive cells in laminae I–III that had a bottom surface within a specified 20- to 30-μm depth of the section, and then determined whether they were nNOS- or NPY-immunoreactive. We then selected NPY-positive cells in the same way and tested whether they were nNOS-immunoreactive.

### Dynorphin-expressing interneurons

Dynorphin is expressed by both inhibitory and excitatory interneurons in the superficial dorsal horn (Sardella et al., 2011a, Duan et al., 2014). To assess the relative frequency and the distribution of these two cell types, we scanned sections from the L4 segments of wild-type mice that had been reacted with antibodies against PPD, Pax2, sst_2A_ and NeuN (three sections per mouse from each segment, *n* = 4 mice). As described above, neurons included in the disector sample were identified and the expression of PPD, Pax2 and sst_2A_ was recorded for each cell. During the course of this analysis, we noted that there was a distinctive mediolateral distribution of the excitatory PPD cells within the dorsal horn, raising the possibility that this was related to sensory inputs from glabrous and hairy skin. We therefore examined the distribution of PPD cells in the L2 segment, which only receives input from hairy skin (Takahashi et al., 2003). A single section reacted to reveal PPD, Pax2 and NeuN was scanned from each of four mice, and the distribution of Pax2^+^ and Pax2^−^ PPD neurons was recorded.

Many of the dynorphin-positive inhibitory interneurons also contain galanin (Brohl et al., 2008, Sardella et al., 2011a), and virtually all galanin-containing cells express the somatostatin receptor sst_2A_ (Iwagaki et al., 2013, Polgar et al., 2013b). In order to assess the extent of colocalization of PPD and galanin, we therefore examined expression of PPD, galanin, sst_2A_ and NeuN in three sections from the L4 segments of each of four mice. Neurons that were immunoreactive for galanin and sst_2A_ were initially selected, and these were then assessed for the presence of PPD. PPD^+^/sst_2A_^+^ neurons that lacked galanin were then identified.

To explore the developmental relationship between dynorphin and nNOS we examined tissue from the L4 segment of Pdyn^Cre^;Ai9 mice. Initially, we quantified the proportion of neurons that were tdTom^+^ in sections reacted to reveal NeuN and PKCγ, and then counterstained with DAPI (*n* = 4 mice), using the disector method. We then determined the extent to which inhibitory and excitatory nNOS cells were labeled with tdTom, by examining sections reacted with antibodies against nNOS and Pax2 (*n* = 2 mice), and testing all nNOS^+^ cells in the section for the presence of Pax2 and tdTom.

In order to characterize neurons that continue to express dynorphin, we examined tissue from close to the injection site in the L4 segment of Pdyn^Cre^;Ai9 mice that had received intraspinal injections of AAV.flex.eGFP. Sections from three mice were reacted with antibodies against PPD and nNOS. TdTom^+^ cells that had their bottom surface within a 20- to 30-μm depth of the section were initially identified, and these were then tested for the presence of GFP, PPD and nNOS.

### Characterization of antibodies

We have shown that immunostaining with the NPY and galanin antibodies is abolished by preincubation with corresponding peptide (Rowan et al., 1993, Simmons et al., 1995). The two antibodies against PPD were raised against a peptide corresponding to amino acids 229–248 at the C terminus of rat PPD, and have been shown to label PPD, but not dynorphin or enkephalin. In addition, immunostaining is blocked by pre-absorption of the antibodies with the immunizing peptide (Lee et al., 1997). The nNOS antibody was raised against the C-terminal 15 amino acids of mouse nNOS. Immunostaining with this antibody co-localizes perfectly with that of a well-characterized nNOS antibody raised in rabbit (AJT, MGM unpublished observations). The parvalbumin antibody is directed against the mouse protein and recognizes a band of 13 kDa on Western blots of mouse brain homogenates (Nakamura et al., 2004). The sst_2A_ antibody was raised against the C terminal 15 amino acids of mouse sst_2A_ receptor coupled to keyhole limpet hemocyanin, and immunostaining is blocked by incubation with the peptide antigen (manufacturer’s specification). The Pax2 antibody was raised against amino acids 188–385 of the mouse protein, and recognizes bands of the appropriate size on Western blots of mouse embryonic kidney (Dressler and Douglass, 1992). The NeuN antibody was raised against cell nuclei extracted from mouse brain and found to react with a protein specific for neurons (Mullen et al., 1992), which has subsequently been identified as the splicing factor Fox-3 (Kim et al., 2009). The PKCγ antibody was raised against a peptide corresponding to the C terminus of mouse PKCγ, and we have shown that it stains identical structures to those labeled with a well-characterized guinea-pig antibody against PKCγ (Sardella et al., 2011b).

### Statistical analysis

The Chi-squared test was used to determine whether nNOS-immunoreactive cells were over-represented among cells that were labeled with tdTomato but not eGFP in the Pdyn^Cre^;Ai9 mice that had been injected with AAV.flex.eGFP. Fisher's exact probability test was used to determine whether the difference in the proportion of PPD neurons that were inhibitory or excitatory differed significantly between the L2 and L4 segments. A *p* value of <0.05 was taken as significant.

## Results

### Quantification of NPY cells

**Fig. 1:**
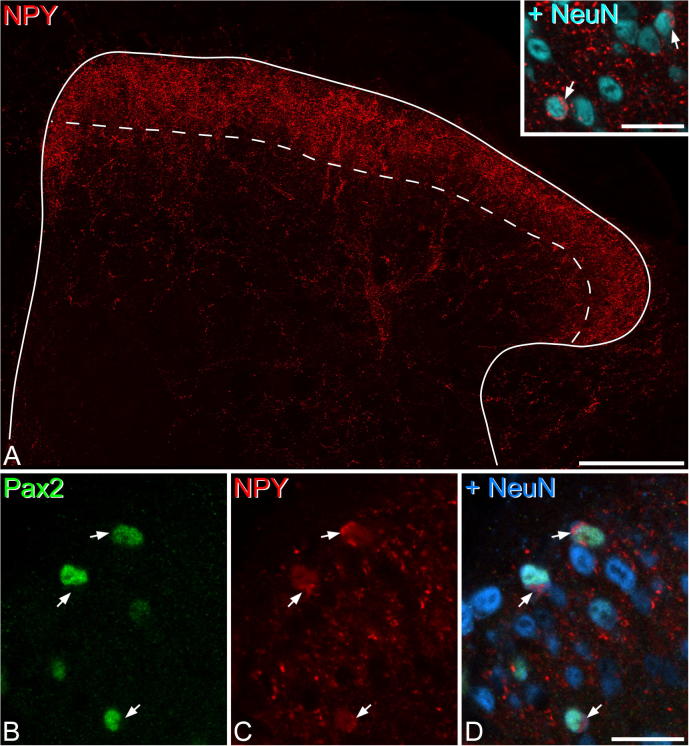
NPY expression in the mouse dorsal horn. (A) A low magnification view of NPY-immunoreactivity in the L4 segment of the mouse dorsal horn. The dashed line represents the border between laminae II and III. There is a plexus of NPY staining, which mostly corresponds to axons, and this is densest in laminae I–II, with scattered axons in deeper laminae. In this image, it is difficult to see NPY-immunoreactive cell bodies, as these are obscured by the axonal plexus. Inset: In a single optical section, NPY-immunoreactive neurons can readily be detected by the presence of immunostaining in the perikaryal cytoplasm. Two of these cells are visible, and are indicated with arrows. (B–D) A single confocal optical section through a section that had been reacted to reveal Pax2 (green), NPY (red) and NeuN (blue). Because the NPY and Pax2 antibodies are both raised in rabbit, the reaction was performed sequentially (for further details see text). Three NPY-expressing cells (arrows) can be recognized by the presence of NPY immunoreactivity in their perikaryal cytoplasm, and in all 3 cases the nucleus is Pax2-positive, indicating that these are inhibitory interneurons. The main image in A is a projection of 30 optical sections at 1-μm z-spacing. Scale bars = 100 μm (main image in A) and 20 μm (B–D and inset in A). (For interpretation of the references to color in this figure legend, the reader is referred to the web version of this article.)

The distribution of NPY-immunoreactivity in the mouse dorsal horn was the same as that reported previously in both mouse and rat (Hunt et al., 1981, Gibson et al., 1984, Rowan et al., 1993, Solway et al., 2011, Iwagaki et al., 2016) (Fig. 1a). Immunoreactive axons formed a dense plexus in laminae I–II, and were more sparsely distributed in deeper laminae. Occasional dense bundles of axons were also seen in laminae III–IV, and it has been shown previously that these are associated with the dendritic trees of projection neurons belonging to the anterolateral tract (Polgár et al., 1999, Cameron et al., 2015). At high magnification, NPY-immunoreactive cell bodies could readily be identified by the immunostaining in their perikaryal cytoplasm (Fig. 1a inset). Quantitative analysis revealed that NPY-immunoreactive cells accounted for 8.4% (range 6.9–9.8%, *n* = 4 mice) and 9.5% (7.1–12.3%) of all neurons in laminae I–II and in lamina III, respectively. In the sections reacted for both NPY and Pax2, NPY-immunoreactivity could easily be detected in the perikaryal cytoplasm of certain neurons, even though Pax2 fluorescence in cell nuclei was also seen in the Rhodamine Red channel due to the sequential staining method (Fig. 1b–d). We identified a total of 265 (range 66–103, *n* = 3 mice) NPY-immunoreactive cells in laminae I–II and 146 (41–55) such cells in lamina III, and found that all of these cells showed Pax2-immunoreactivity in their nuclei. Although we did not analyze the deeper dorsal horn laminae (IV–VI), we did observe a few NPY cells that lacked Pax2 in this region.

We have previously reported that inhibitory (GABA-immunoreactive) cells account for 25.8% of all neurons in laminae I–II and 37.6% of those in lamina III (Polgar et al., 2013a). We therefore estimate that NPY-immunoreactive cells account for 33% of the inhibitory interneurons in laminae I–II and for 25% of those in lamina III.

### Colocalization of NPY with dynorphin, sst_2A_, parvalbumin and nNOS

**Fig. 2:**
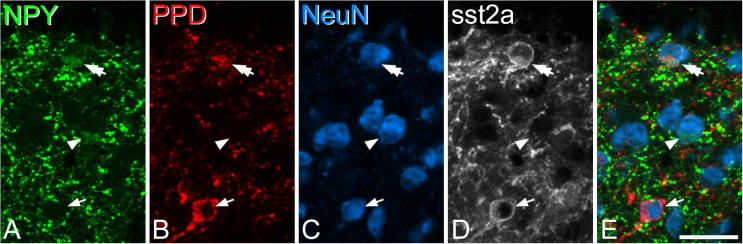
Co-expression of neuropeptide Y (NPY) and preprodynorphin (PPD) in a lamina II neuron. A scan through laminae I and II from a section that had been reacted with antibodies against neuropeptide Y (NPY, green), preprodynorphin (PPD, red), NeuN (blue) and sst_2A_ (gray). (A) Two NPY-immunoreactive cells are indicated (arrowhead and double arrow) and in each case, the immunoreactivity is present in the perikaryal cytoplasm. (B) The cell marked with the double arrow, and another cell (indicated with an arrow) are PPD-immunoreactive. (C) Staining with the NeuN antibody shows that these three cells are all neurons. (D) sst_2A_ is expressed by the 2 PPD-positive neurons (arrow, double-arrow), but not by the cell that was NPY-positive and PPD-negative (arrowhead). (E) A merged image, shows the relationship between NPY-, PPD- and NeuN-immunoreactivities. The images represent projections of 3 optical sections at 1-μm z-spacing. Scale bar = 20 μm. (For interpretation of the references to color in this figure legend, the reader is referred to the web version of this article.)

In the sections reacted to reveal NPY, PPD, sst_2A_ and NeuN, we restricted the analysis of colocalization to laminae I–II, because inhibitory PPD cells are infrequent in deeper laminae. We identified 192 NPY^+^ cells (range 32–71, *n* = 4 mice) and 277 PPD^+^ cells (52–87) in laminae I–II. We found that 13.1% (5.3–19.7%) of the NPY-immunoreactive cells also contained PPD immunoreactivity, and this corresponded to 9.3% (3.6–16.9%) of the PPD^+^ cells. Among the neurons with immunoreactivity for both peptides 20 out of 27 (74%) cells were sst_2A_^+^ (Fig. 2).

**Fig. 3:**
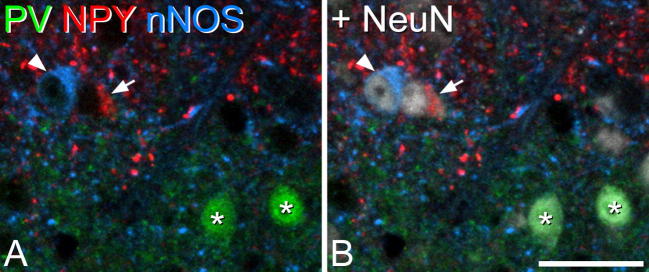
Lack of overlap between cells that express NPY, parvalbumin (PV) and nNOS in the inner part of lamina II. (A) A single optical section from tissue that had been scanned to reveal PV (green), NPY (red) and nNOS (blue). (B) The same optical section, but with immunostaining for NeuN shown in gray. Four cells have been highlighted: two of these (*) are immunoreactive for PV, one (arrow) for NPY and one (arrowhead) for nNOS. Staining for NPY and nNOS is in the perikaryal cytoplasm, and the nucleus of these cells can be seen due to immunostaining for NeuN in (B). Scale bar = 20 μm. (For interpretation of the references to color in this figure legend, the reader is referred to the web version of this article.)

In sections reacted to reveal NPY, nNOS and parvalbumin (Fig. 3), we identified a total of 121 parvalbumin-immunoreactive neurons in laminae I–II (range 20–59, *n* = 3 mice), and 366 (89–139) such cells in lamina III, reflecting the much higher density of parvalbumin cells in deeper laminae, compared to the superficial dorsal horn, seen in the mouse (Hughes et al., 2012). Quantitative analysis revealed that in laminae I–II there were 4.8 parvalbumin cells per 10 μm length of the L4 segment. This length of the mouse L4 segment would be expected to contain 124.8 neurons, of which 25.8% (i.e. 32.2 neurons) are inhibitory (Polgar et al., 2013a). We have shown that 75% of parvalbumin neurons in laminae I–II are inhibitory in the rat (Laing et al., 1994) and a similar proportion has been seen for parvalbumin neurons in laminae I–III of the mouse (Abraira et al., 2017). Based on this, we estimate that parvalbumin cells account for ∼11% of the inhibitory interneurons in laminae I–II. None of the parvalbumin cells in laminae I–II and only 1 out of 366 parvalbumin cells in lamina III were nNOS-immunoreactive. There was very limited overlap between parvalbumin and NPY: 4 out of 121 (3.3%) of the parvalbumin cells in laminae I–II, and 7 out of 366 (1.9%) of those in lamina III were NPY-immunoreactive. However, since this overlap is likely to be restricted to inhibitory parvalbumin cells (which are thought to correspond to 75% of all parvalbumin neurons), the extent of overlap is probably somewhat higher for these cells: 4.3% in laminae I–II and 2.5% in lamina III. To determine the extent of overlap of NPY and nNOS, we identified 247 NPY-positive cells (71–101) in laminae I–II and 140 (43–49) in lamina III. We found that 4.4% of those in laminae I–II and 6.4% of those in lamina III were also nNOS-immunoreactive.

### Expression of preprodynorphin and its relation to galanin

**Fig. 4:**
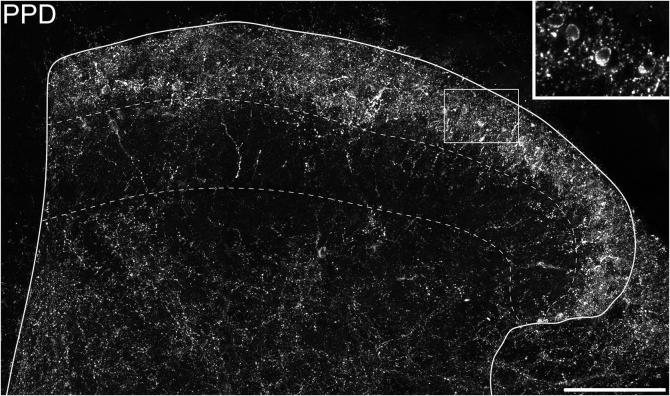
Preprodynorphin (PPD) immunoreactivity in the mouse dorsal horn. Confocal image of a transverse section through the L4 segment, reacted with antibody against PPD. A dense band of immunostaining is present in lamina I and the dorsal part of lamina II, with variable extension into the ventral part of lamina II. This region contains several immunoreactive cell bodies, and these are seen more clearly in the inset (corresponding to the box in the main image), in which four immunoreactive cells are visible. Lamina III contains a few scattered immunoreactive axons and cell bodies, while immunoreactive axons are more numerous in laminae IV–V. Dashed lines represent the dorsal and ventral borders of lamina III. The main image is a projection of 30 optical sections and the inset is a projection of three sections. In each case the z-spacing is 1 μm. Scale bar = 100 μm.

**Fig. 5:**
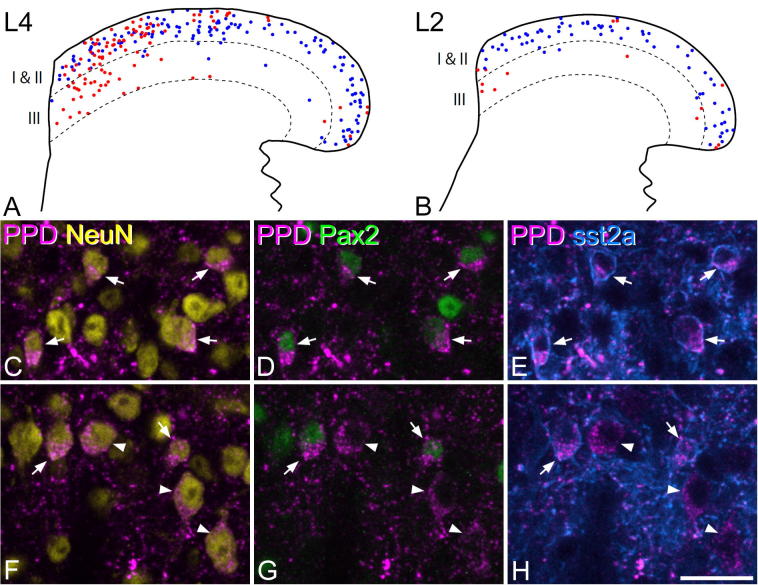
The distribution of inhibitory and excitatory neurons that express preprodynorphin (PPD) in mouse lumbar spinal cord. (A) The distribution of PPD-immunoreactive cells identified in the L4 segments of four mice and plotted onto an outline of the dorsal horn. Pax2-negative (excitatory) neurons are shown in red and Pax2-positive (inhibitory) neurons are shown in blue. The upper and lower dashed lines represent the borders of lamina III. Note that although inhibitory PPD neurons are evenly distributed throughout the superficial dorsal horn, the excitatory cells are clustered in the medial one third. (B) A corresponding plot for the distribution of PPD-immunoreactive cells in the L2 segment of four mice shows that there are very few excitatory (Pax2-negative) cells, and these are scattered throughout the superficial laminae. (C–E) Confocal images from the middle part of lamina II in the L4 segment scanned to reveal PPD (magenta), NeuN (yellow), Pax2 (green) and sst_2A_ (blue). Four PPD-immunoreactive neurons are indicated with arrows, and all of these are also immunoreactive for both Pax2 and sst_2A_, indicating that they are all inhibitory interneurons. (F–H) Corresponding confocal images from the medial part of lamina II in L4, scanned in the same way. This field includes two PPD cells with both Pax2 and sst_2A_ (arrows), as well as three PPD cells that lack both Pax2 and sst_2A_ (arrowheads) and are therefore excitatory interneurons. All confocal images are projections of two optical sections at 1-μm z-spacing. Scale bar (applies to all confocal images) = 20 μm. (For interpretation of the references to color in this figure legend, the reader is referred to the web version of this article.)

In sections reacted to reveal PPD, the distribution of immunostaining was similar to that seen in the rat (Sardella et al., 2011a), with a dense plexus in the superficial dorsal horn and scattered profiles in the deeper laminae (Fig. 4). PPD-immunoreactive neurons were present throughout the dorsal horn, but they were most numerous in laminae I–II. In the sections analyzed from the L4 segment, PPD was detected in 203 lamina I–II neurons (range 41–67 *n* = 4 mice), and these corresponded to 11.3% (9.9–12.6%) of all neurons in laminae I–II. The majority of these cells (67%, range 54–76%) were Pax2-immunoreactive, and could therefore be identified as inhibitory interneurons. The PPD^+^/Pax2^+^ cells accounted for 30.8% (25.7–35.2%) of the Pax2 cells in lamina I–II. The great majority (91%, range 84.4–100%) of the inhibitory PPD cells were sst_2A_, and these accounted for 54% (48.2–58.2%) of the sst_2A_-immunoreactive cells. Interestingly, we found that although the inhibitory PPD cells were uniformly distributed across the mediolateral extent of the dorsal horn, the excitatory (Pax2-negative) PPD cells showed a highly uneven distribution, being largely restricted to the medial one third (Fig. 5a, c–h). Within this medial region we also observed numerous Pax2-negative PPD cells in lamina III. The medial part of the L4 segment receives inputs from primary afferents that innervate glabrous skin, raising the possibility that dynorphin-expressing excitatory interneurons are predominantly associated with this type of input. To test this, we also examined sections from the L2 segment, which is only innervated by afferents from hairy skin (Takahashi et al., 2003). We identified PPD-positive cells in a 20-μm disector from 1 section in each of four mice and determined whether these cells were Pax2-immunoreactive. As predicted, we found a quite different pattern, with a far higher proportion of PPD-immunoreactive cells being Pax2-positive (90%, 66/73 PPD cells in laminae I–II, data pooled from four mice). Unlike the situation in L4, there was no clustering of PPD^+^/Pax2^−^ cells in the medial part of laminae I–II, although a few excitatory cells were present in the medial part of lamina III (Fig. 5b). The difference in the proportion of inhibitory and excitatory PPD cells between the L4 and L2 segments was highly significant (Fisher's exact probability test, *p* < 0.0001).

**Fig. 6:**
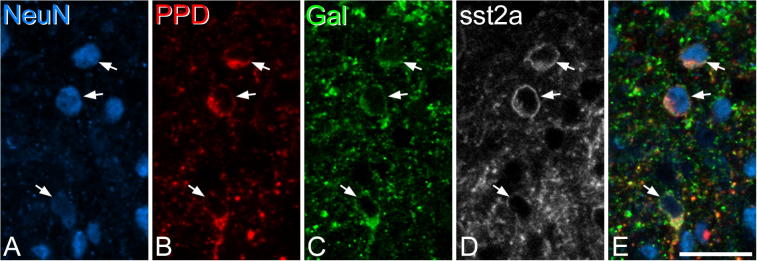
Co-expression of preprodynorphin (PPD), galanin (gal) and sst_2A_ in lamina II neurons. A scan through the superficial dorsal horn from a section that had been reacted with antibodies against NeuN (blue), PPD (red), galanin (green) and sst_2A_ (gray). (A–D) Three NeuN-positive cells that are immunoreactive for PPD, galanin and sst_2A_ are indicated with arrows. (E) A merged image, showing NeuN, PPD and galanin. The images are from a single optical section. Scale bar = 20 μm. (For interpretation of the references to color in this figure legend, the reader is referred to the web version of this article.)

In sections reacted with antibodies against galanin, PPD, sst_2A_ and NeuN we restricted the analysis to neurons that were sst_2A_-immunoreactive, because virtually all galanin neurons (Iwagaki et al., 2013) and the great majority of inhibitory PPD neurons (see above) express this receptor. We identified 256 (range 59–72, *n* = 4 mice) galanin^+^/sst_2A_^+^ neurons and 288 (64–79) PPD^+^/sst_2A_^+^ neurons per mouse. The vast majority (95.8%, range 92.2–100%) of galanin-immunoreactive cells were also PPD^+^, and galanin was present in 85% (range 81–92%) of the PPD^+^/sst_2A_^+^ cells (Fig. 6).

### Pdyn^Cre^;Ai9 mouse

**Fig. 7:**
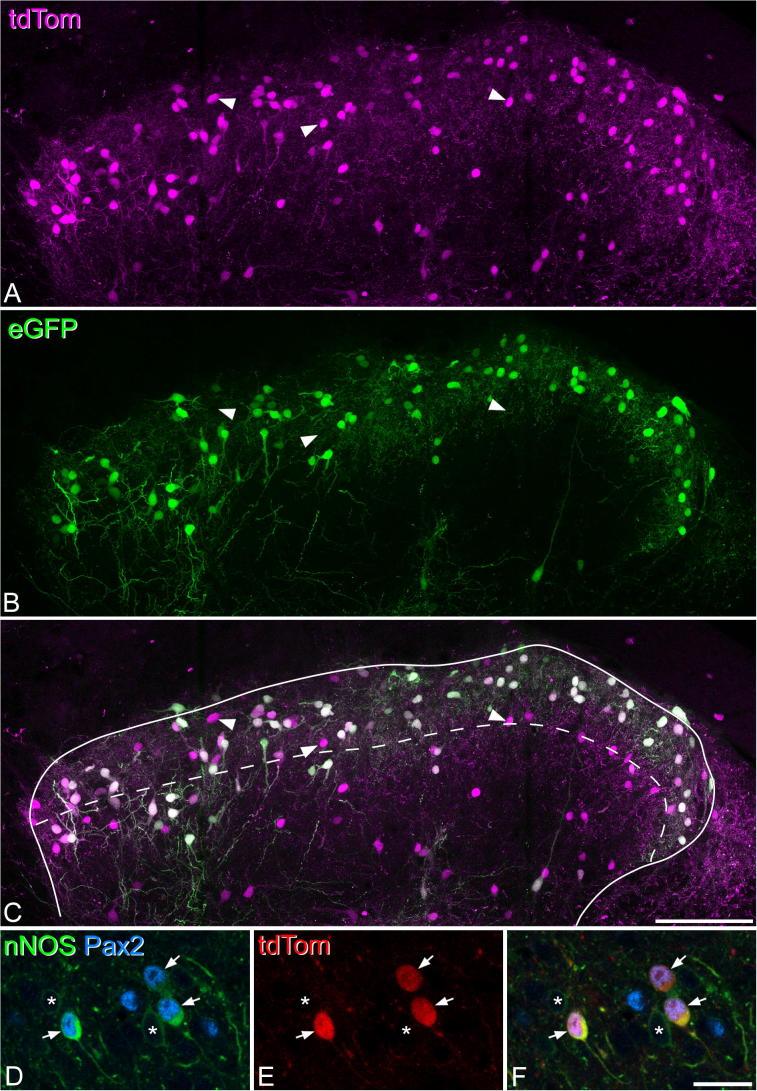
Expression of enhanced green fluorescent protein (eGFP) and tdTomato (tdTom) in a Pdyn^Cre^;Ai9 mouse that had received an intraspinal injection of AAV.flex.eGFP at P44. (A) tdTom-positive cells (magenta) are particularly numerous in laminae I–II, but are scattered throughout the remainder of the dorsal horn. (B, C) eGFP (green) is expressed in many, but not all of the tdTom cells, and three cells that lack eGFP are indicated with arrowheads. Note that virtually all of the eGFP cells also express tdTom. The dashed line indicates the lamina II–III border. (D–F) A single optical section through a Pdyn^Cre^;Ai9 mouse that had been reacted to reveal nNOS (green) and Pax2 (blue). Three nNOS-positive cells with Pax2-immunoreactive nuclei are shown with arrows. These correspond to nNOS-expressing inhibitory interneurons, and all three are tdTom-positive. Two other nNOS-positive cells that lack Pax2 are also visible (*) and these cells, which correspond to nNOS-expressing excitatory neurons, lack tdTom. Note that the excitatory nNOS cells show much weaker immunoreactivity for nNOS. The images in A–C were obtained from 45 optical sections at 1-μm z-spacing. Scale bar = 100 μm (A–C), 20 μm (D–F). (For interpretation of the references to color in this figure legend, the reader is referred to the web version of this article.)

In the sections from the Pdyn^Cre^;Ai9 mice, tdTom-positive neurons were seen throughout the dorsal horn, but were present at a relatively high density in laminae I–II (Fig. 7a). Between 63 and 112 tdTom^+^ neurons were identified in each of the four mice (two sections per mouse), and these cells constituted 13.9% (range 11.5–17.7%) of all neurons in laminae I–II, and 4.3% (2.6–6.3%) of those in lamina III. Two sections each from two mice were analyzed for expression of nNOS and Pax2. We identified a total of 164 nNOS-expressing inhibitory interneurons (i.e. cells that were both nNOS- and Pax2-immunoreactive) in laminae I–III in these sections (81, 83 in each mouse) and found that 82% of these cells (77%, 86%) were tdTom-positive (Fig. 7d–f). We also identified 299 nNOS-expressing excitatory (Pax2-negative) neurons (152, 147 in each mouse) and found that only one of these cells (0.3%) contained tdTomato. This indicates that most inhibitory nNOS cells express dynorphin at some stage during their development, but that this is not the case for excitatory nNOS cells.

**Fig. 8:**
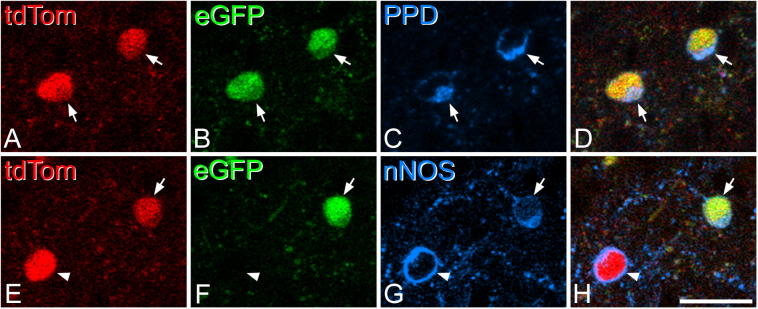
Expression of enhanced green fluorescent protein (eGFP) and tdTomato (tdTom) in neurons that contain preprodynorphin (PPD) or neuronal nitric oxide synthase (nNOS) in a Pdyn^Cre^;Ai9 mouse that had received an intraspinal injection of AAV.flex.eGFP at P44. (A–D) Two cells that express both tdTom (red) and eGFP (green) are immunoreactive for PPD (blue) and are indicated with arrows. (E–H) This field shows two tdTom-expressing cells that are immunoreactive for nNOS (blue). One of these (arrow) is positive for eGFP, while the other (arrowhead) is not. All images are from single optical sections. Scale bar = 20 μm. (For interpretation of the references to color in this figure legend, the reader is referred to the web version of this article.)

We analyzed two sections from each of the three mice that received intraspinal injection of AAV.flex.eGFP, and found that 79% (range 71–84%) of the tdTom^+^ neurons in laminae I–II in the region near the injection site contained eGFP, indicating that they continued to express dynorphin past the early postnatal period (Fig. 7a–c). The lack of eGFP in the remaining 21% of tdTom^+^ neurons could have resulted from either lack of infection by the AAV, or because these cells no longer expressed dynorphin. As expected, virtually all (243/245, 99%) of the eGFP-containing cells were tdTom^+^. The results of the neurochemical analysis of eGFP-positive and eGFP-negative tdTom cells were similar across the three mice, and data were therefore pooled (Table 2). Among tdTom^+^ cells in laminae I–II, 72% were PPD-immunoreactive, whereas among eGFP-containing cells the proportion rose to 85%. The lack of detectable PPD-immunoreactivity in 15% of eGFP cells may have resulted from the lower sensitivity of the TSA-Cy5 kit, which had to be used in this case because the TSA-tetramethylrhodamine product could not be distinguished from tdTom fluorescence. The great majority (93%) of the PPD-immunoreactive tdTom^+^ cells contained eGFP (Fig. 8a–d), and the small number that did not contain eGFP probably results from lack of infection of these cells by the injected AAV. Nonetheless, these results indicate that PPD can be detected in the cell bodies of the great majority of cells that continue to express dynorphin, as defined by the presence of eGFP. Interestingly, we found that a smaller proportion of the nNOS-immunoreactive tdTom^+^ cells (40/69, 58%) contained eGFP compared to the remaining (nNOS-negative) tdTom^+^ cells (203/240, 85%) (Fig. 8e–h). This difference was highly significant (Chi-squared test, *p* < 0.0001), indicating that nNOS^+^ tdTom cells were under-represented among those that continue to express dynorphin. This suggests that although most inhibitory nNOS cells in laminae I–III initially express dynorphin, many of these cells cease to do so during development.

**Table 2:** PPD- and nNOS-immunoreactivity and eGFP expression among tdTom-positive cells in the Pdyn^Cre^ mice injected with AAV.flex.eGFP

	eGFP+				eGFP-			
	PPD+/nNOS+	PPD+/nNOS−	PPD-/nNOS+	PPD-/nNOS−	PPD+/nNOS+	PPD+/nNOS−	PPD-/nNOS+	PPD-/nNOS−
	**26** (3–12)	**180** (57–64)	**14** (3–7)	**23** (5–9)	**4** (1–3)	**12** (0–10)	**25** (4–13)	**25** (2–18)

Total	**243**				**66**			

## Discussion

The main findings of this study are: (1) that NPY-immunoreactivity is restricted to inhibitory interneurons in laminae I–III, but is present in a far higher proportion (33%) of these cells than we found in the rat; (2) that PPD expression largely overlaps with that of galanin, and shows ∼10% co-localization with NPY; (3) that as in the rat, NPY, nNOS and parvalbumin inhibitory interneuron populations show little or no overlap; and (4) that although the distribution of inhibitory PPD cells in laminae I–II appears uniform, the pattern is quite different for excitatory PPD cells, which are clustered in the medial third of the dorsal horn at the L4 level, corresponding to the region innervated from glabrous skin.

### Neurochemical populations among inhibitory interneurons in laminae I–II

The results of this study, taken together with quantitative data from our previous studies (Iwagaki et al., 2013, Polgar et al., 2013a) allow us to estimate the sizes of the various neurochemical populations that we have identified among inhibitory interneurons in the superficial dorsal horn of the mouse spinal cord.

As noted above, our findings suggest that parvalbumin cells account for around 11% of inhibitory interneurons in laminae I–II. This population shows essentially no overlap with nNOS cells, and only ∼4% of the parvalbumin cells express NPY (corresponding to 0.5% of all inhibitory neurons). Although we did not directly compare galanin and parvalbumin it is unlikely that there is a significant co-expression, because parvalbumin cells are highly concentrated in lamina IIi–V (Hughes et al., 2012, Abraira et al., 2017), while the galanin cells are largely restricted to laminae I–IIo (Tiong et al., 2011, Iwagaki et al., 2013).

We have previously provided quantitative data concerning inhibitory interneurons that express galanin and/or nNOS in laminae I–II of the mouse (Iwagaki et al., 2013). We reported that all galanin cells were sst_2A_-positive, and that these accounted for 44% of the sst_2A_ cells. Since sst_2A_ is expressed by 54% of inhibitory interneurons in laminae I–II (Polgar et al., 2013a), this means that galanin can be detected in ∼24% of inhibitory neurons in this region. Similarly, nNOS was present in 30% of the sst_2A_ cells, and since 95% of inhibitory nNOS cells are sst_2A_^+^, nNOS cells would correspond to 17% of the inhibitory neurons. However, there is a moderate degree of overlap between galanin and nNOS in the mouse. Galanin and/or nNOS neurons make up 61% of the sst_2A_ cells, and if we allow for the small number of inhibitory nNOS neurons that lack sst_2A_, then we estimate that the combined populations constitute 34% of all inhibitory cells in laminae I–II.

From the present results, we conclude that NPY can be detected in 33% of the inhibitory cells in the superficial dorsal horn, and this is considerably higher than our estimate (18%) for the corresponding population in the rat (Polgár et al., 2011). Although there may be a significant species difference in the proportion of inhibitory interneurons that express NPY, a more likely explanation is that in the mouse, cells that synthesize NPY have higher levels of the peptide in their perikaryal cytoplasm, meaning that more of them reach the detection threshold for immunocytochemistry. We found that 13% of NPY cells were PPD^+^, and three-quarters of these were sst_2A_-immunoreactive. We had previously reported that 24% of NPY cells in the mouse were sst_2A_-immunoreactive (Iwagaki et al., 2013), and the present results show that around two-fifths of these cells also express dynorphin. We did not directly investigate co-localization of NPY and galanin, because the two antibodies that we used to detect these peptides were both raised in rabbit. However, our findings with PPD and NPY provide an indication of the likely extent of co-expression. Since galanin is found in most dynorphin-expressing inhibitory neurons, but is restricted to those with sst_2A_, ∼10% of the NPY cells are likely to co-express galanin, and this would correspond to 3.3% of all inhibitory neurons. Similarly, nNOS was detected in 4.4% of NPY cells, which would equate to ∼1.5% of the inhibitory cells.

**Fig. 9:**
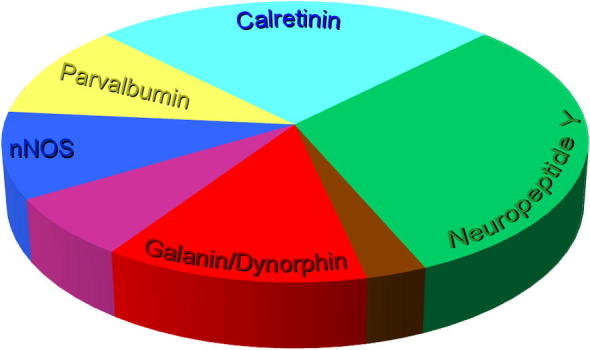
Proportions of inhibitory interneurons in laminae I–II that belong to different neurochemical populations. The pie chart shows the estimated proportions of all inhibitory interneurons in laminae I–II that are accounted for by each neurochemical population. There is considerable overlap between galanin/dynorphin (red) and nNOS (blue) populations, shown in purple. Similarly there is limited overlap between galanin/dynorphin cells and the NPY population (green) and this is shown in brown. Note that there is also a small overlap of the NPY population with both nNOS and parvalbumin cells, and this is not shown on the pie chart. (For interpretation of the references to color in this figure legend, the reader is referred to the web version of this article.)

Based on these numbers, we estimate that cells that contain one or more of these four neurochemical markers make up ∼75% of the inhibitory (GABAergic) interneurons in laminae I–II of the mouse dorsal horn. Interestingly, Smith et al. (2015) recently identified a novel population of calretinin-expressing inhibitory cells, which constituted ∼25% of the Pax2-positive neurons in this region, and which showed very little overlap with the NPY, nNOS or galanin populations that we describe here. In addition, we have found that they do not overlap with the parvalbumin-immunoreactive cells (AJT and M Mustapa, unpublished observations). This means that most, if not all, inhibitory interneurons in laminae I–II are likely to belong to one of these five neurochemical populations (Fig. 9).

The opioid peptide enkephalin is expressed by many neurons in laminae I–III (Todd, 2017). These include both excitatory and inhibitory interneurons (Huang et al., 2010, Francois et al., 2017), and some of the enkephalin cells also express nNOS or parvalbumin (Huang et al., 2010). However, both of these proteins are also found in excitatory cells, and it is not yet known whether the inhibitory enkephalinergic neurons correspond to one or more of the classes that we define here.

### Functions of the different neurochemical populations

Among these populations, the function is probably best understood for the parvalbumin cells, which are found in lamina IIi and the deep dorsal horn (Antal et al., 1990, Laing et al., 1994, Hughes et al., 2012, Abraira et al., 2017). Hughes et al. (2012) demonstrated that these cells received monosynaptic input from low-threshold myelinated (A-LTMR) afferents, and that their axons frequently formed axoaxonic synapses onto A-LTMR afferents, which suggests that they play an important role in inhibiting tactile input. Direct evidence for this was subsequently provided by Petitjean et al. (2015), who showed that ablating parvalbumin neurons led to mechanical hypersensitivity, while activating them increased mechanical pain thresholds and alleviated tactile allodynia in a neuropathic pain model.

We have previously demonstrated that the galanin/dynorphin and nNOS inhibitory interneuron populations both depend on the transcription factor Bhlhb5 during development, and we therefore refer to these as B5-I neurons (Chiang et al., 2016). Since *Bhlhb5*^−^*^/^*^−^ mice show chronic itch, we concluded that the B5-I neurons include cells that suppress itch in response to counterstimuli (Kardon et al., 2014). Because dynorphin acts on the κ opioid receptor (KOR), and intrathecally administered KOR agonists suppressed itch, we also proposed that loss of KOR signaling (secondary to depletion of dynorphin) was likely to contribute to the exaggerated itch seen in *Bhlhb5*^−^*^/^*^−^ mice (Kardon et al., 2014). There is evidence that neuropeptides can initially be expressed in a relatively large number of superficial dorsal horn neurons during development, with expression being switched off in some of these cells (Duan et al., 2014, Gutierrez-Mecinas et al., 2017). It has also been shown that expression of nNOS in this region occurs relatively late during development, with nNOS-expressing cells reaching their definitive number in the 3rd week after birth in the rat (Liuzzi et al., 1993). We had therefore speculated that some B5-I cells might switch from neuropeptide to nNOS expression during early development (Iwagaki et al., 2013). Our findings in the Pdyn^Cre^ mouse confirm this prediction, since nNOS-expressing inhibitory interneurons were over-represented among the tdTom^+^/eGFP^−^ neurons (i.e. “transient dynorphin” cells), following injection of AAV.flex.eGFP. Duan et al. (2014) used the same Pdyn^Cre^ line with an intersectional strategy that allowed them to ablate only the inhibitory dynorphin neurons. However, the present results show that they would probably have affected more than just those inhibitory interneurons that continue to express dynorphin in the adult. This ablation resulted in a dramatic increase in mechanical pain, indicating a major role for the B5-I neurons in suppressing transmission of noxious mechanical input to dorsal horn projection neurons.

Although the main purpose of this study was to investigate inhibitory interneurons, we also made a surprising observation concerning the distribution of Pax2-negative PPD neurons. These cells, most of which are presumably excitatory interneurons (Sardella et al., 2011a), were far more numerous in the medial one-third of the L4 segment than in the remaining parts of this segment, or in L2, and this presumably reflects an association with sensory inputs from glabrous skin. Interestingly, although we had reported an almost complete loss of PPD-immunoreactive cells in the *Bhlhb5*^−^*^/^*^−^ mouse (Kardon et al., 2014), Duan et al. (2014) subsequently found a much smaller reduction in the number of PPD mRNA cells (∼50%) in the same knockout. Our analysis of dynorphin neurons was made in the L3 segment, which receives little input from glabrous skin (Takahashi et al., 2003), and would therefore likely have contained predominantly inhibitory PPD neurons. Although Duan et al. do not indicate which segment(s) they analyzed, the PPD mRNA cells that they illustrate in the *Bhlhb5*^−^*^/^*^−^ mouse are highly concentrated in the medial part of the dorsal horn (their Fig. S7L). If this image was taken from the L4 or L5 segment, these could be the excitatory PPD neurons. These cells are likely to be retained in the *Bhlhb5*^−^*^/^*^−^ mouse, as there is no evidence for loss of excitatory neurons in this genotype (Ross et al., 2010), and we have observed numerous PPD-immunoreactive neurons that lack Pax2 in the medial part of the L5 segment in these mice (MGM, KAB, AJT unpublished observations).

Relatively little is known about the functions of NPY-expressing interneurons. NPY itself can act on Y1 and Y2 receptors, which are widely expressed on dorsal horn neurons and central terminals of primary afferents, and the majority of studies have reported that NPY has an antinociceptive effect (Brumovsky et al., 2007, Smith et al., 2007). However, although knockdown of NPY prolonged neuropathic or inflammatory pain, it had little effect on acute nociceptive thresholds, suggesting that under physiological conditions, NPY is only antinociceptive in chronic pain states (Solway et al., 2011). Since the NPY-expressing cells account for nearly a third of GABAergic neurons in laminae I–II and around a quarter of those in lamina III, they are likely to make an important contribution to GABAergic inhibitory mechanisms in the dorsal horn. Many of these cells respond to noxious stimuli (Polgar et al., 2013b; Iwagaki et al., 2016), which suggests that they are involved in antinociception. A recent study by Bourane et al. (2015) used a bacterial artificial chromosome transgenic mouse, in which Cre is expressed under control of the NPY promoter to investigate the function of NPY neurons in the dorsal horn. They reported that ablation of Cre-expressing cells evoked a form of mechanical itch in hairy skin, but caused no alterations in responses to painful stimuli. This is surprising, given the widespread distribution of NPY-expressing cells and axons, which are present throughout the mediolateral extent of the dorsal horn, including regions that receive input from glabrous skin. However, only ∼35% of the cells that were targeted by Bourane et al. were positive for NPY mRNA in adult mice, suggesting that many of them were transient NPY expressers. It is therefore not clear to what extent the population that they ablated corresponds to the NPY-immunoreactive inhibitory interneurons that we describe. Further studies will be needed to assess the roles of NPY-expressing inhibitory interneurons.

## Conclusion

The present results show that expression of galanin/dynorphin, NPY, nNOS and parvalbumin defines four largely non-overlapping populations that account for ∼75% of the inhibitory interneurons in laminae I–II of the mouse dorsal horn. When taken together with a recently identified population of calretinin-expressing cells, our findings suggest that virtually all of the inhibitory neurons in the superficial dorsal horn can be assigned to one of these neurochemical classes.
